# Phylogenetic Relationships in *Pterodroma* Petrels Are Obscured by Recent Secondary Contact and Hybridization

**DOI:** 10.1371/journal.pone.0020350

**Published:** 2011-05-31

**Authors:** Ruth M. Brown, William C. Jordan, Chris G. Faulkes, Carl G. Jones, Leandro Bugoni, Vikash Tatayah, Ricardo L. Palma, Richard A. Nichols

**Affiliations:** 1 Institute of Zoology, Zoological Society of London, London, United Kingdom; 2 School of Biological and Chemical Sciences, Queen Mary University of London, London, United Kingdom; 3 Mauritian Wildlife Foundation, Vacoas, Mauritius; 4 Durrell Wildlife Conservation Trust, Trinity, Jersey, United Kingdom; 5 Institute of Biomedical and Life Sciences, University of Glasgow, Glasgow, United Kingdom; 6 Instituto de Ciências Biológicas, Fundação Universidade Federal do Rio Grande, Rio Grande, Brazil; 7 Museum of New Zealand Te Papa Tongarewa, Wellington, New Zealand; The University of Queensland, St. Lucia, Australia

## Abstract

The classification of petrels (*Pterodroma* spp.) from Round Island, near Mauritius in the Indian Ocean, has confounded researchers since their discovery in 1948. In this study we investigate the relationships between Round Island petrels and their closest relatives using evidence from mitochondrial DNA sequence data and ectoparasites. Far from providing clear delimitation of species boundaries, our results reveal that hybridization among species on Round Island has led to genetic leakage between populations from different ocean basins. The most common species on the island, *Pterodroma arminjoniana*, appears to be hybridizing with two rarer species (*P. heraldica* and *P. neglecta*), subverting the reproductive isolation of all three and allowing gene flow. *P. heraldica* and *P. neglecta* breed sympatrically in the Pacific Ocean, where *P. arminjoniana* is absent, but no record of hybridization between these two exists and they remain phenotypically distinct. The breakdown of species boundaries in Round Island petrels followed environmental change (deforestation and changes in species composition due to hunting) within their overlapping ranges. Such multi-species interactions have implications not only for conservation, but also for our understanding of the processes of evolutionary diversification and speciation.

## Introduction

Molecular phylogenies are now commonly used to disentangle relationships among taxa that have proven difficult to classify by other means. The gadfly petrels (*Pterodroma* spp.) provide an excellent example; the taxonomic treatment of this group has provoked considerable debate among researchers and is frequently revised, with traditional classification methods based on phenotype, anatomy and calls often proving insufficient to differentiate species. Molecular phylogenetic analysis provides a logical alternative, and molecular methods have already proven useful in determining the identity of some disputed *Pterodroma* species [1]. In this study we focus on petrels which breed at Round Island (22 km NE of Mauritius in the Indian Ocean) and their relationship to congeners that breed worldwide. Since their discovery in 1948 [2], the origin and classification of Round Island petrels has been uncertain. The aim of this investigation is to clarify the taxonomic treatment of *Pterodroma* on Round Island, and to determine the relationships between these birds and a number of closely related species, whose taxonomy is controversial.

**Figure 1:**
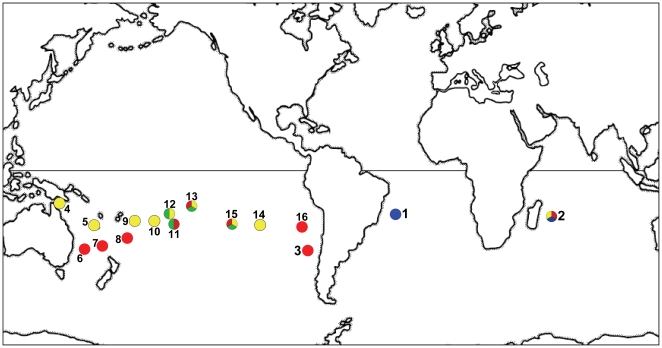
Distribution of selected *Pterodroma* petrels. *P. arminjoniana* (blue^1, 2^), *P. neglecta* (red^2, 3, 6, 7, 8, 11, 13, 15, 16^), light morph *P. heraldica* (yellow^2, 4, 5, 9, 10, 12, 13, 14, 15^) and *P. atrata*/dark morph *P. heraldica* (green^11, 12, 13, 15^). ^1^Trindade Island, ^2^Round Island, ^3^Juan Fernandez, ^4^Raine Island, ^5^New Caledonia, ^6^Lord Howe Island, ^7^Phillip Island, ^8^Kermadec Islands, ^9^Tonga, ^10^Cook Islands, ^11^Australs, ^12^French Polynesia, ^13^Tuamotus, ^14^Easter Island, ^15^Pitcairn Islands (including Henderson and Ducie), ^16^Desventuradas.

At least three species of *Pterodroma* have been recorded breeding on Round Island (Fig. 1), and historical records suggest that extensive breeding only became established within the last century. The fauna and flora of Round Island have been documented by visiting naturalists since 1844 [3], and a variety of seabird species have been recorded on the island. However, it was not until the mid 1940s that breeding petrels were first unambiguously reported [2]. The Round Island population was initially identified as a single species, *P. arminjoniana* [4], but in the mid 1980s a second petrel species, *P. neglecta*, was also discovered to be breeding there, though in much smaller numbers than *P. arminjoniana* [5]. Since the mid 1990s, small, very pale petrels with a white ventral surface and a greyish head have been recorded at Round Island which might be a third species, *P. heraldica* (C. Jones *pers. obs.*). One of these small petrels has been clearly identified as *P. heraldica* from banding data. This bird was caught on Round Island in April 2006 and its band number confirmed it as a *P. heraldica* banded on Raine Island, Australia (Fig. 1), in July 1984 [6]. Prior to their discovery on Round Island, the range of *P. arminjoniana* was thought to be restricted to the Atlantic Ocean, where it breeds on a single island (Trindade Island, 1200 km east of the Brazilian coast) and the ranges of *P. heraldica* and *P. neglecta* were thought to be restricted to the Pacific Ocean, where they breed sympatrically, in some cases on the same island [7] (Fig. 1). The presence of a breeding population of *P. neglecta* on Trindade Island has been suggested in a single paper by Imber [8]. However, Imber's evidence is derived largely from second-hand sources and is highly questionable. A convincing rebuttal of Imber's conclusion has been published by Tove [9]. In addition, L. Bugoni spent a considerable amount of time on Trindade Island and has examined numerous live birds in the field. He found no birds with the pale primary shafts characteristic of *P. neglecta*, nor did he hear any *neglecta*-type calls on the island. Current evidence therefore suggests that *P. neglecta* are not present on Trindade Island.

The current taxonomic treatment of gadfly petrels regards *P. arminjoniana*, *P. neglecta* and *P. heraldica* as separate species [10]. However, with no single characteristic that unambiguously identifies these species, there has been considerable disagreement over their classification in the past. Murphy [11] suggested that *P. arminjoniana* should be regarded as a subspecies of *P. neglecta*. These taxa are very similar in appearance; both are polymorphic and display a similar range of plumage types, from an entirely dark form through to a pale form which has a white abdomen and face. There are, however, some notable differences between the two taxa, namely that *P. neglecta* is larger with a shorter, rounder tail and has characteristic white shafts on the primary flight feathers [12]. The calls of these two species are also strikingly different. As a result, Murphy's taxonomic treatment was not adopted and Murphy returned these taxa to full species in a later revision of the group [13].

In their 1952 paper on the taxonomy of the *Pterodroma*, Murphy & Pennoyer [13] lumped *P. arminjoniana* together with another Pacific species, *P. heraldica*, giving them both sub-specific status. Some authors do not even go this far, instead referring to *P. arminjoniana* and *P. heraldica* as a single species with no designated subspecies (eg. [6]). The two species are similar in appearance and have almost identical calls, though *P. heraldica* are smaller than *P. arminjoniana*. On at least one island where they breed (Henderson), *P. heraldica* do not display polymorphism, but are all pale morph birds [7], whereas *P. arminjoniana* are polymorphic at all their known breeding localities. In his extensive revision of the taxonomy of gadfly petrels, Imber [14] restored both *P. heraldica* and *P. arminjoniana* to full species, a decision based largely on intestinal structure and feather lice hosted by the birds. Imber found that the intestinal structure of *P. arminjoniana* was identical to that of *P. neglecta*, but differed from that of *P. heraldica*. In addition, *P. arminjoniana* from Trindade Island and *P. neglecta* both host the same louse species, *Halipeurus kermadecensis*, whereas *P. heraldica* hosts a different species, *H. heraldicus*. Intriguingly, there have been reports that the petrel population on Round Island, thought to comprise mainly *P. arminjoniana* and *P. neglecta*, also hosts *H. heraldicus* [15]. Lice are relatively immobile and do not survive away from their host, therefore dispersal between individuals occurs via direct contact between hosts, usually during copulation or care of the young [16]. A newly introduced louse species is thought to be capable of replacing the pre-existing species if the latter is reduced in numbers, or absent in the case of founding hosts arriving without lice [17]. *Pterodroma* petrels are host to seven genera of chewing lice (order Phthiraptera), including eight species from the genus *Halipeurus* [18]. These *Halipeurus* form two distinctive species groups, the ‘marquesanus’ group containing two species and the ‘procellariae’ group containing six species [14]. The differences between these two louse species-groups are so great that Timmermann [19] proposed classifying the gadfly petrels into two distinct genera based on the species-group of louse that they hosted, although this proposal was never adopted. *H. kermadecensis* belongs to the ‘procellariae’ lice group whilst *H. heraldicus* belongs to the ‘marquesanus’ lice group, and the two can be easily distinguished from each other by the genitalia and the relative length of the abdominal segments in males, and by the shape of the terminalia in females [20]. Although 29 species of *Halipeurus* are known to parasitize 78 species and subspecies of petrels [18], there is only a single recorded example of two species of *Halipeurus* simultaneously infesting the same host population [21], supporting the idea of rapid species displacement in lice. The presence of a different louse species on *P. arminjoniana* and *P. neglecta* from Round Island and their supposed parental populations in the Atlantic and Pacific Oceans would therefore suggest a host-switching event has taken place at some time in the past.

A fourth petrel species, *P. atrata*, may also be considered part of the *neglecta/heraldica/arminjoniana* complex. *P. atrata* is a dark coloured Pacific petrel that breeds sympatrically with *P. heraldica* on Henderson Island. Originally considered to be polymorphic variants of the same species, *P. heraldica* and *P. atrata* were split in 1995 on the basis of assortative mating, mitochondrial sequence data and analysis of calls [7]. The dark morph of *P. heraldica* has been recorded on a number of other Pacific Islands and these birds may also be *P. atrata*, though this remains to be confirmed.

Petrels on Round Island can be tentatively classified on the basis of their size and plumage characteristics. Polymorphic birds with dark primary shafts are most similar to *P. arminjoniana* [5] and these dark-shafted birds make up the majority of the population on Round Island. Birds with white primary shafts most closely resemble *P. neglecta* [12] and are present in smaller numbers, making up around 10% of the population [5]. In addition small, pale birds, which may be *P. heraldica*, appear to be present on the island in similar numbers to the white-shafted birds (C. Jones *pers. obs.*). Field observations on Round Island indicate that mixed-species pairs are not uncommon. Pairs of dark-shafted and white-shafted birds have been observed on a number of occasions, and at least one of these pairs has successfully hatched a chick [22]. The bird identified as *P. heraldica* from its band number was caught whilst rearing a chick, and appeared to be paired with a dark-shafted bird resembling *P. arminjoniana*. There are also birds present on the island that are intermediate between the morphological extremes of these species, and birds giving calls that appear intermediate between the calls of *P. neglecta* and *P. arminjoniana/heraldica*. These observations suggest that hybridization is occurring on Round Island between *P. arminjoniana* and *P. neglecta* and also between *P. arminjoniana* and *P. heraldica*. Analysis of microsatellite genotype data from Round Island petrels and from the Trindade Island population of *P. arminjoniana* has confirmed that *P. arminjoniana* and *P. neglecta* are hybridizing at Round Island. Genetic analysis of a suspected hybrid chick and its parents (a female *P. neglecta* and a male *P. arminjoniana*) showed that these birds were indeed the true parents of the chick. Allele frequency data also indicated a high degree of admixture between *P. arminjoniana* and *P. neglecta* on Round Island [22].

In this study, we aim to expand the genetic assessment of Round Island and Trindade Island petrels using sequence data from the mitochondrial gene cytochrome-*b*, and to include *P. heraldica* from Round Island and additional populations of *P. heraldica*, *P. neglecta* and *P. atrata* from the Pacific Ocean in the analysis. We aim to explore whether mitochondrial sequence data can be used to delimit species boundaries in this group of petrels, and attempt to determine whether hybridization on Round Island involves all three species of petrels present on the island. We will also examine a larger sample of feather lice than any previous study, collected from birds on Trindade and Round Islands, to provide additional support for our hypothesis of secondary contact and hybridization between species on Round Island. The role of deforestation in creating suitable breeding habitat for petrels on Round Island, and thus leading to the contact and hybridization among three petrel species, is discussed.

## Methods

### Ethics statement

There are no legal restrictions covering research on animals in Mauritius, however all animal work carried out during this study was conducted in accordance with UK Home Office guidelines. The species sampled are not listed by CITES (Convention on the International Trade in Endangered Species). Biological samples were exported from Mauritius under a memorandum of agreement between the Government of Mauritius and Queen Mary University of London. Samples were imported into the UK under DEFRA import licence number AHZ/2295/2004/1. Fieldwork techniques involving live animals were approved by an ethical review committee at the Institute of Zoology, Zoological Society of London (project code GFA/0383).

### Phylogenetic analysis

We sequenced a 995 base pair (bp) fragment of the mitochondrial gene cytochrome-*b* (cyt-*b*) for 21 *P. arminjoniana* from Trindade Island and 26 *P. arminjoniana* ( = birds with dark primary shafts), 11 intermediate birds ( = birds with intermediate phenotype), 8 *P. neglecta* ( = birds with white primary shafts) and 1 *P. heraldica* (identified from banding data) from Round Island using polymerase chain reaction (PCR) and automated DNA sequencing. The entire cyt-*b* gene (∼1100 bp) was amplified as a single fragment using the PCR primers L14863 [23] and H15965 (5′-GTGAGGGAAGCTA GTTGACCG-3′). Amplifications were performed in a 30 µl reaction mix containing ∼10 ng genomic DNA; 1× PCR buffer; 2.9 mM MgCl_2_; 146 µM each of dATP, dCTP, dGTP and dTTP; 4.38 µM of each primer, ∼14 µg BSA and 3 units of *Taq* DNA polymerase (Invitrogen). Thermal cycling was carried out in a GeneAmp PCR System 9700 (Applied Biosystems) and consisted of 1 min at 95°C then 40 cycles of 1 min at 94°C, 1 min at 45°C, 1 min at 63°C and 3 min at 72°C with a final cycle of 5 min at 72°C. PCR products were visualised in 2% agarose gel containing 0.3 µg/ml ethidium bromide. Fragments of length 1100 bp were cut directly from the gel and purified using a QIAquick Gel Extraction Kit (Qiagen). Sequencing was carried out using 5 µl of purified product in 15 µl volume cycle-sequencing reactions. The reaction mix contained 1 µl of BigDye Terminator v3.1 Cycle Sequencing Solution (Applied Biosystems); 5 µl Better Buffer (Microzone Ltd) and 0.16 µM of primer. The thermal cycle consisted of 96°C for 3 min followed by 30 cycles of 96°C for 15 s, 50°C for 10 s and 60°C for 4 min with a final step of 60°C for 5 min. Sequencing products were purified using ethanol precipitation, resuspended in 10 µl HiDi Formamide (ABI) and visualised on an ABI 3100 Automated DNA Sequencer. Initial sequence data produced using the external primers was used to design internal sequencing primers L15238 (5′- CAGGAGTTATACTTCTACTTACCC-3′), L15551 (5′- CATTCCACCCCTACTTCACCC-3′), H15533 (5′- GATACGATACC GAGAGGGTTG-3′), H15158 (5′- GAGGCTCCGTTTGCATGTAGGTTT-3′). Subsequent sequencing reactions using all six primers were carried out as above and produced six overlapping fragments spanning 995 bp of the cyt-*b* gene. Sequences were aligned and edited using Sequencher 4.1 (Applied Biosystems) and Bioedit 7.0.5 [24]. Cytochrome-*b* sequences produced during this study are deposited with GenBank, accession numbers GQ328969–GQ328988. Also included in the analysis were published cyt-*b* sequences for 60 petrels from the Pacific Ocean. One complete cyt-*b* sequence was downloaded from Genbank (U74341, *P. neglecta* from Juan Fernandez). 307 bp fragments of cyt-*b* for 10 *P. neglecta* from the Kermadec Islands and 32 *P. heraldica* and 17 *P. atrata* from the Pitcairn Islands were acquired from [5] and [7] (data not available on Genbank).

Phylogenetic relationships were estimated using maximum likelihood and Bayesian optimality criteria. Four outgroup taxa were included (*P. externa* U74339, *P. phaeopygia* U74340, *P. inexpectata* U74346, *P. solandri* U74347). The model of nucleotide evolution used was the HKY85 model [25] with a Gamma distribution of substitution rates (shape parameter = 0.0001). This model was chosen as having the best fit to the data using modeltest 3.7 [26]. Maximum likelihood analysis was conducted using paup 4.0b10 [27], with bootstrap resampling to assess support for internal branches. Multiple heuristic searches with random addition of taxa were carried out to minimise the effect of input order bias. Branch swapping used the tree bisection-reconnection (TBR) algorithm and bootstrapping was performed with 1000 replicates. Bayesian analyses were conducted using mrbayes 3.1 [28], [29] with default priors. The nucleotide substitution type (nst) was set at 2, corresponding with the HKY model of nucleotide substitution. A gamma model of rate variation across sites was invoked using the rates = invgamma function. Analyses were initiated with random starting trees and run for 1 million generations with trees sampled every 100 generations. The first 250,000 generations (2500 trees) were discarded as burn-in and posterior probabilities were estimated from the remaining sampled generations. Two separate analyses with two independent chains were carried out and the log-likelihood values were compared to check that the chains had converged and were mixing well. Maximum likelihood analysis produced 34 trees with equal likelihood. These trees were combined in a 50% majority-rule consensus tree, which had a similar topology to the tree produced using Bayesian estimation. A likelihood ratio test indicated that sequences were evolving in a clock-like manner (−log*L*_no clock_ = 2167.48; −log*L*_clock_ = 2170.26; *P*>0.05). Average sequence divergence within and between phylogroups was calculated from Nei's corrected average pairwise differences [30] using arlequin 2.0 [31], based on the 307 bp fragment for which all individuals were sequenced. A statistical parsimony network for all haplotypes was estimated using a 95% confidence limit with the program tcs v1.21 [32].

### Feather lice

28 feather lice from Round Island (collected from 21 dark-shafted, 2 intermediate and 5 white-shafted birds), and 217 feather lice from Trindade Island (collected from 77 *P. arminjoniana*) were identified to species by R.L.P.

## Results

### Phylogenetic analysis

**Figure 2:**
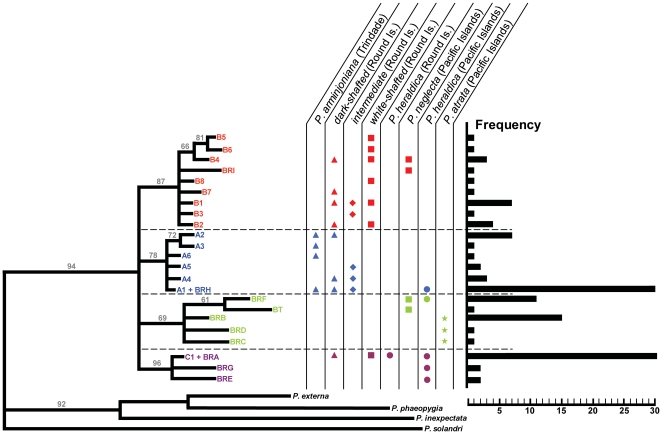
Bayesian phylogenetic tree based on cyt-*b* haplotypes. Four distinct phylogroups (red, blue, green, purple) are visible. Posterior probability support values are shown above branches. Average sequence divergence between phylogroups ranges from 0.70% to 1.35%. A1–A6, B1–B8 and C1 sequenced during this study; BRA–BRI from [5]; BT from [7]. Columns show distribution of haplotypes between species/populations (▴ = *P. arminjoniana* (or dark-shafted birds), ▪ = *P. neglecta* (or white-shafted birds) • = *P. heraldica*, ★ = *P. atrata*, ♦ = intermediate). Frequency of each haplotype is also shown.

Twenty-three unique haplotypes were identified within the data set (Table 1). Haplotypes sequenced during this study were named A1–A6, B1–B8 and C1. The *P. neglecta* haplotype downloaded from Genbank was identical to haplotype B4 sequenced during this study. Haplotypes from [5] and [7] were named A–I and T following the original publications. Two of the short haplotype fragments published by [7] were identical to the corresponding section of two longer haplotypes sequenced during this study. These matching long and short haplotypes were therefore considered to be identical. A mitochondrial DNA-based phylogenetic tree for the Round Island birds, together with data from petrels originating from the historical ranges of *P. arminjoniana*, *P. neglecta* and *P. heraldica*, revealed four distinct phylogroups within the data set (Fig. 2). The phylogeny provides evidence of a genetic contribution from three species (*P. arminjoniana*, *P. neglecta* and *P. heraldica*) into the Round Island population. Atlantic *P. arminjoniana* haplotypes (blue phylogroup) are shared with dark-shafted and intermediate birds from Round Island. Pacific *P. neglecta* haplotypes (red phylogroup) are shared with dark-shafted, intermediate and white-shafted Round Island birds. Similarly, one Pacific *P. heraldica* haplotype (purple phylogroup) is shared with haplotypes from dark-shafted and white-shafted birds, and the *P. heraldica* sampled on Round Island.

**Table 1:** Distribution of 23 unique haplotypes from *Pterodroma* petrels.

Haplotype	*n*	*P. arm*Trindade Island	*dark- shafted* Round Island	*intermediate*Round Island	*white-shafted* Round Island	*P. her*Round Island	*P. neg* Pacific Islands	*P. her* Pacific Islands	*P. atrata*Pacific Islands
**A1+BRH**	**30**	17	7	5				1*	
**A2**	**7**	2	5						
**A3**	**1**	1							
**A4**	**3**		2	1					
**A5**	**2**			2					
**A6**	**1**	1							
**B1**	**7**		4	2	1				
**B2**	**4**		2		2				
**B3**	**1**			1					
**B4+U74341**	**3**		1		1		1**		
**B5**	**1**				1				
**B6**	**1**				1				
**B7**	**1**		1						
**B8**	**1**				1				
**C1+BRA**	**30**		4		1	1		24*	
**BRB**	**15**								15*
**BRC**	**1**								1*
**BRD**	**1**								1*
**BRE**	**2**							2*	
**BRF**	**11**						8†	3*	
**BRG**	**2**							2*	
**BRI**	**1**						1†		
**BT**	**1**						1†		
**Total**	**127**	**21**	**26**	**11**	**8**	**1**	**11**	**32**	**17**

A1–A6, B1–B8, C1 sequenced during this study; BRA–BRI from [5]; BT from [7]; U74341 downloaded from GenBank.

*Pitcairn Islands.

**Juan Fernandez.

† Kermadec Islands.

A single *P. heraldica* from the Pacific (Ducie Island) was found to have a haplotype (BRH) identical to the most common haplotype found in *P. arminjoniana* from Trindade (A1). Some *P. heraldica* from Ducie Island also shared a haplotype with *P. neglecta* from the Kermadecs (BRF).

**Figure 3:**
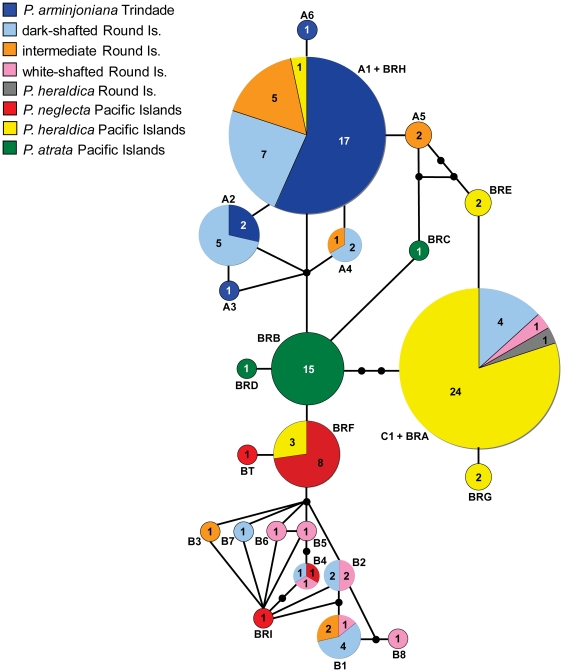
Statistical parsimony network of haplotypes from Round Island, Trindade and Pacific populations of *Pterodroma*. Circles are proportional to the total number of individuals showing each haplotype, haplotype name is given next to each circle, coloured areas show the proportion of each haplotype assigned to each population, numbers show number of individuals. Connecting lines represent a single base substitution and small filled circles represent hypothetical unsampled haplotypes.

The complex relationship between *P. arminjoniana*, *P. neglecta*, *P. heraldica* and *P. atrata* can be visualised using a mitochondrial-DNA haplotype network (Fig. 3). Haplotypes from dark-shafted Round Island birds (pale blue) are spread throughout the network, clustering with Trindade *P. arminjoniana* (dark blue), Pacific *P. neglecta* (red), Pacific *P. heraldica* (yellow) and white-shafted Round Island birds (pink). However, haplotypes from Atlantic *P. arminjoniana* (dark blue) are restricted to one area of the network, as are haplotypes from Pacific *P. neglecta* (red). *P. atrata* haplotypes (green) are not shared with any other group and are centrally located in the network, suggesting that they are ancestral to the other haplotype groups.

### Feather lice

All lice collected from petrels on Round Island were identified as *Halipeurus heraldicus* and all those collected from petrels on Trindade Island were identified as *H. kermadecensis*.

## Discussion

Molecular phylogenetic analysis of *Pterodroma* petrels from Round Island, Trindade and the Pacific Islands does not reveal clear differentiation among the currently recognized species. Given the differences in phenotype, anatomy, calls and ectoparasites among these taxa, as well as the uneven distribution of haplotypes, it seems unlikely that they represent a single, polytypic species complex. Therefore the observed pattern of haplotype distribution might be due to shared ancestral polymorphism and incomplete lineage sorting, or to hybridization between previously distinct lineages.

On Round Island, birds with dark primary shafts have been observed breeding with birds that have white primary shafts. Analysis of microsatellite genotype data suggests that hybridization is occurring between dark- and white-shafted birds [22], and in this study we show that dark- and white-shafted birds on Round Island also share three mitochondrial DNA haplotypes. The presumed parental populations of the Round Island birds (*P. arminjoniana* from Trindade and *P. neglecta* from the Pacific Islands) do not share any haplotypes, therefore we conclude that the overlapping haplotypes on Round Island are the result of hybridization between these two species rather than shared ancestral polymorphism.

The single *P. heraldica* caught on Round Island was observed breeding with a dark-shafted, dark morph bird presumed to be *P. arminjoniana*. The mtDNA haplotype of this *P. heraldica* was identical to haplotypes found in both dark-shafted and white-shafted Round Island birds. It was also the most common haplotype found in *P. heraldica* from the Pacific Islands, but was not found in either *P. arminjoniana* from Trindade or *P. neglecta* from the Pacific. Again, these results suggest that hybridization on Round Island, rather than ancestral polymorphism, is responsible for haplotype sharing between species.

Some haplotypes recorded in the Round Island petrel population were not seen in any of the presumed parental populations of *P. arminjoniana*, *P. neglecta* or *P. heraldica*. It seems unlikely that these haplotypes have arisen by mutation within the Round Island population, given the population size and time available. A more plausible explanation for is that the parental populations have been incompletely sampled.

Feather lice collected from *P. arminjoniana* (dark-shafted) and *P. neglecta* (white-shafted) on Round Island were identified as a single species, *H. heraldicus*, whereas *P. arminjoniana* from Trindade were host to *H. kermadecensis*. Identification of feather lice from Round Island birds therefore suggests contact between *P. heraldica* and the other species present on the island, with consequent infestation of *P. arminjoniana* and *P. neglecta* by *H. heraldicus*. Co-phylogenetic analysis of *Halipeurus* lice and their hosts has confirmed that the presence of *H. heraldicus* on *P. arminjoniana* and *P. neglecta* on Round Island is the result of a host switch whereas *H. kermadecensis* is the ancestral parasite of *P. arminjoniana* [15].

The data presented here are consistent with the hypothesis that multi-species hybridization on Round Island has led to leakage of genetic material between previously isolated populations of *P. arminjoniana*, *P. neglecta* and *P. heraldica* from the Atlantic and Pacific Oceans. Field observations, evidence from microsatellite genotypes and identification of feather lice together indicate close contact and hybridization between these species on Round Island. In addition, analysis of mtDNA haplotypes reveals a high degree of haplotype sharing by the three species on Round Island. Whilst, in theory, the mixture of haplotypes on Round Island could be due to retained ancestral polymorphism, the reciprocal monophyly of putative parental populations from different ocean basins argues that hybridisation is a more parsimonious explanation. This interpretation is strongly reinforced by the independent evidence of hybridisation at nuclear loci, obtained for the two taxa for which ancestral frequencies could be estimated [22] and direct observations of successful breeding by pairs with the distinct phenotypes.

The colonization of Round Island by petrels appears to have followed habitat alterations on the island. Human-induced environmental degradation on Round Island is well documented [33], [34]. The original hardwood forest was destroyed by introduced goats and rabbits, much of the topsoil washed away, and poaching reduced the numbers of some bird species. Today, although goats and rabbits have been eradicated, poaching has ceased and vegetation is being restored, the island consists largely of bare rock. Both *P. arminjoniana* and *P. neglecta* prefer to nest on exposed, rocky hill-sides, therefore deforestation and reduced competition with other bird species would have increased the number of suitable nest sites available for these species on Round Island. This newly available nesting habitat may have been instrumental in the sudden appearance of petrels on the island.

Round Island petrels may represent a rare example of multispecies hybridization in naturally occurring vertebrate populations. The implications of this discovery are two-fold. Firstly, ongoing environmental degradation is predicted to alter the ranges of species and increase the likelihood of secondary contact, which could in turn result in a cascade of genetic homogenization involving multiple taxa. The situation on Round Island supports this prediction. Secondly, our results demonstrate that multispecies reticulate evolution can occur in natural animal systems and that some species may have genomes that are a mosaic of three or more ancestral taxa. Such reticulate evolutionary events have received attention from a number of authors recently [35]–[38] and may be more widespread in nature than previously thought. The commonly used tree-like model of evolution and speciation may therefore not always be realistic, and alternative methods of phylogenetic reconstruction are required which can further uncover and explore reticulate events.

## References

[1] Zino F, Brown RM, Biscoito M (2008). The separation of *Pterodroma madeira*
(Zino's Petrel) from *Pterodroma feae* (Fea's
Petrel) (Aves: Procellariidae).. Ibis.

[2] Vinson J (1949). L'ile Ronde et l'ile aux serpents.. Proc Roy Soc Arts Sci Mauritius.

[3] Lloyd JA (1846). Letter read to the Society on the subject of Round & Serpent
Islands.. Proc-Verbeaux, Soc Nat Hist Mauritius.

[4] Rountree FRG, Guerin R, Pelte S, Vinson J (1952). Catalogue of the birds of Mauritius.. Bull Mauritius Inst.

[5] Brooke MDeL, Imber MJ, Rowe G (1999). Occurrence of two surface-breeding species of
*Pterodroma* on Round Island, Indian
Ocean.. Ibis.

[6] King BR, Reimer DS (1991). Breeding and behaviour of the Herald Petrel *Pterodroma
arminjoniana* on Raine Island, Queensland.. Emu.

[7] Brooke MdeL, Rowe G (1995). Behavioural and molecular evidence for specific status of light
and dark morphs of the Herald Petrel *Pterodroma
heraldica*.. Ibis.

[8] Imber MJ (2004). Kermadec petrels (*Pterodroma neglecta*) at Ilha
da Trindade, South Atlantic Ocean and in the North Atlantic.. Notornis.

[9] Tove MH (2005). Kermadec Petrels (*Pterodroma neglecta*) in the
Atlantic Ocean – a rebuttal.. Notornis.

[10] Brooke M (2004). Albatrosses and Petrels across the World.

[11] Murphy RC (1936). Oceanic Birds of South America.

[12] Onley D, Scofield P (2007). Field Guide to the Albatrosses, Petrels and Shearwaters of the
World.

[13] Murphy RC, Pennoyer JM (1952). Large petrels of the Genus
*Pterodroma*.. Amer Mus Nov.

[14] Imber MJ (1985). Origins, phylogeny and taxonomy of the gadfly petrels
*Pterodroma* spp.. Ibis.

[15] Hammer S, Brown RM, Bugoni L, Palma RL, Hughes J (2010). On the origin of *Halipeurus heraldicus* on Round
Island petrels: Cophylogenetic relationships between petrels and their
chewing lice.. Mol Phyl Evol.

[16] Marshall AG (1981). The Ecology of Ectoparasitic Insects.

[17] Paterson AM, Palma RL, Gray RD, Page RDM (2003). Drowning on arrival, missing the boat and
*x*-events: how likely are sorting events?. Tangled Trees. Phylogeny, Cospeciation, and Coevolution.

[18] Price RD, Hellenthal RA, Palma RL, Johnson KP, Clayton DH (2003). The Chewing Lice: World Checklist and Biological
Overview.. Illinois Natural History Survey Special Publication.

[19] Timmermann G (1965). Die Federlingsfauna der Sturmvogel und die Phylogenese des
procellariiform Vogelstammes.. Abhandlungen und Verhandlungen des Naturwissenschaftlichen Vereins in
Hamburg.

[20] Timmermann G (1960). Gruppen-Revisionen bei Mallophagen. II. Genus
*Halipeurus* Thompson 1936. 1. Teil: Die Halipeurus-Arten
der “gadfly-petrels” (Genera *Pterodroma* und
*Bulweria*).. Zeitschrift für Parasitenkunde.

[21] Palma RL, Imber MJ (2000). Coexistence of two species of *Halipeurus*
(Phthiraptera) on Chatham Island Taiko (*Pterodroma
magentae*) (Aves).. N Z J Zool.

[22] Brown RM, Nichols RA, Faulkes CG, Jones CG, Bugoni L (2010). Range expansion and hybridization in Round Island petrels
(*Pterodroma arminjoniana*); evidence from microsatellite
genotypes.. Mol Ecol.

[23] Nunn GB, Cooper J, Jouventin P, Robertson CJR, Robertson GG (1996). Evolutionary relationships among extant albatrosses
(Procellariiformes: Diomedeidae) established from complete cytochrome-b gene
sequences.. The Auk.

[24] Hall TA (1999). BioEdit: a user-friendly biological sequence alignment editor and
analysis program for Windows 95/98/NT.. Nuc Acids Symp Ser.

[25] Hasegawa M, Kishino H, Yano T-A (1985). Dating of the human-ape splitting by a molecular clock of
mitochondrial DNA.. J Mol Evol.

[26] Posada D, Crandall KA (1998). Modeltest: testing the model of DNA substitution.. Bioinformatics.

[27] Swofford DL (2002). PAUP* Phylogenetic Analysis Using Parsimony (*and other
methods). Version 4.

[28] Ronquist F, Huelsenbeck JP (2003). MrBayes 3: Bayesian phylogenetic inference under mixed
models.. Bioinformatics.

[29] Ronquist F, Huelsenbeck JP, van der Mark P (2005). MrBayes 3.1 Manual.. http://mrbayes.csit.fsu.edu/manual.php.

[30] Nei M, Li W-H (1979). Mathematical model for studying genetic variation in terms of
restriction endonucleases.. Proc Nat Acad Sci USA.

[31] Schneider S, Roessli D, Excoffier L (2000). Arlequin ver. 2.0: A software for population genetics data
analysis.. http://lgb.unige.ch/arlequin/.

[32] Clement M, Posada D, Crandall KA (2000). TCS: a computer program for estimating gene
genealogies.. Mol Ecol.

[33] Bullock DJ, North S (1977). Round Island: a tale of destruction.. Oryx.

[34] Cheke AS, Hume J (2008). Lost Land of the Dodo.

[35] Linder CR, Rieseberg LH (2004). Reconstructing patterns of reticulate evolution in
plants.. Am J Bot.

[36] Seehausen O (2004). Hybridization and adaptive radiation.. Trends in Ecol Evol.

[37] Gompert Z, Fordyce JA, Forister ML, Shapiro AM, Nice CC (2006). Homoploid hybrid speciation in an extreme
habitat.. Science.

[38] McDonald DB, Parchman TL, Bower MR, Hubert WA, Rahel FJ (2008). An introduced and a native vertebrate hybridize to form a genetic
bridge to a second native species.. Proc Natl Acad Sci USA.

